# Dioscin Alleviates Periodontitis by Inhibiting NLRP3 Inflammasome Activation via Regulation of K^+^ Homeostasis and Mitochondrial Function

**DOI:** 10.7150/ijbs.85851

**Published:** 2024-01-27

**Authors:** Xue Jiang, Xinxin Ding, Jianxu Wei, Xiaolei Lv, Yi Zhang, Yijie Yang, Hongchang Lai, Xiaomeng Zhang

**Affiliations:** Department of Oral and Maxillofacial Implantology, Shanghai Ninth People's Hospital, Shanghai Jiao Tong University School of Medicine; College of Stomatology, Shanghai Jiao Tong University; National Center for Stomatology; National Clinical Research Center for Oral Diseases; Shanghai Key Laboratory of Stomatology; Shanghai Research Institute of Stomatology, Shanghai 200011, China.

**Keywords:** Periodontitis, Dioscin, NLRP3 inflammasome, mtROS, ox-mtDNA, K^+^ efflux.

## Abstract

Gingival inflammation and alveolar bone loss are characteristic manifestations of periodontitis. Interleukin (IL)-1β, the maturation of which is mainly regulated by NOD-like receptor protein (NLRP) 3 inflammasome, not only amplifies the inflammatory response but also triggers osteoclastogenesis, thereby accelerating the progression of periodontitis. Dioscin, a natural steroid saponin, has been shown to inhibit NLRP3 inflammasome. Nevertheless, research on the effectiveness of Dioscin for the management of periodontitis remains scarce. In this study, Dioscin was found to dramatically reduce the integral components of NLRP3 inflammasome, ultimately limiting IL-1β secretion. Notably, the inhibitory impact of Dioscin on NLRP3 inflammasome might be exerted by curbing the generation of mitochondrial (mt) reactive oxygen species (ROS) and oxidized (ox) mtDNA, which were mediated by inhibition of K^+^ efflux. Furthermore, Dioscin effectively alleviated periodontitis in mice. Overall, the results established that Dioscin could alleviate periodontitis by inhibiting NLRP3 inflammasome via modulation of the K^+^ efflux-mtROS-ox-mtDNA pathway, holding the potential to treat periodontitis and other NLRP3-driven inflammatory diseases.

## Introduction

As is well documented, periodontitis damages the gingiva tissue, periodontal ligament, and alveolar bone, and even leads to tooth loss 1. In 2019, 1.1 billion people worldwide were estimated to suffer from severe periodontitis 2. An imbalance between the host immune system of the host and the microbial community contributes to the initiation and progression of periodontitis 3. Existing treatment strategies involving mechanical therapy and antibiotics aim at minimizing bacterial load. However, the use of antibiotics may trigger side effects and demonstrate limited efficacy in bone regeneration 1. Thus, it is urgent to discover novel therapeutic targets to inhibit periodontitis progression efficiently.

Interleukin (IL)-1β, predominantly secreted by macrophages, not only recruits immune cells to amplify the inflammatory response 4 but also triggers the degradation of collagen by increasing matrix metalloproteinases secretion 5 and promotes nuclear factor kappa B (NF-κB) ligand (RANKL) mediated osteoclastogenesis 6, eventually resulting in bone resorption in periodontitis. IL-1β is transformed from the inactive precursor pro-IL-1β and subsequently proteolytically processed into the functional IL-1β by Caspase-1, a component of the NOD-like receptor protein (NLRP) 3 inflammasome 7. NLRP3 inflammasome has been reported to play an instrumental role in the pathology of inflammatory diseases and is regarded as the therapy target for these diseases 8-10. According to a previous study, NLRP3 deficiency and the administration of NLRP3 inhibitors could efficiently suppress alveolar bone loss by inhibiting IL-1β maturation 11. Besides, clinical trials have revealed that NLRP3 and IL-1β levels in saliva and gingival tissues are higher in periodontitis patients and are correlated with the severity of periodontitis 12, 13. Based on these findings, it is postulated that governing NLRP3 inflammasome might be effective in the management of periodontitis.

Research focusing on natural phytochemicals is emerging owing to their broad range of biological activities, low toxicity, cost-effectiveness, and easy availability. Among them, Dioscin, a natural steroid saponin 14, could inhibit oxidative stress 15 and inflammation 16, 17, as well as facilitate osteogenesis 18-20. Dioscin could alleviate inflammation by inhibiting NLRP3 inflammasome in NLRP3-related diseases such as colitis 21 and acute brain injuries 22. In lipoteichoic acid (LTA)-induced inflammatory macrophages, Dioscin curtailed the production of reactive oxygen species (ROS) and phosphorylation of NF-κB, leading to a downregulation in NLRP3 expression 20. Thus, it is hypothesized that Dioscin could be used to manage periodontitis by modulating the NLRP3 inflammasome. However, there is limited evidence about its therapeutic effect in periodontitis and its underlying mechanism remains elusive.

The activation process of the NLRP3 inflammasome comprises priming and activation steps 23. The former, induced by lipopolysaccharide (LPS), facilitates mRNA expression of *Nlrp3* and *Il1β* through NF-κB pathway. The latter, stimulated with extracellular ATP, pore-forming toxins, etc., thereby drives mitochondrial (mt) dysfunction 24, 25 and concurrently impairs ion homeostasis 26, 27, resulting in the assembly of NLRP3 components, namely NLRP3 inflammasome activation. According to earlier reports, Dioscin suppresses NF-κB activation 20 and mtROS generation 28. Hence, it is speculated that Dioscin could inhibit NLRP3 inflammasome by governing both priming and activation processes.

The protective effect of Dioscin against inflammation was explored by detecting the mRNA and protein expression of pro-inflammatory cytokines. Afterward, the role of Dioscin on NLRP3 inflammasome was examined. Finally, the effectiveness of Dioscin in alleviating alveolar bone resorption was further assessed in mice models.

## Materials and Methods

### Reagents

Dioscin was sourced from Chengdu Must Bio-technology Co., Ltd. *Porphyromonas gingivalis*-LPS was procured from InvivoGen. The ROS Assay Kit, Mito-Tracker Red CMXRos, Hoechst 33342 staining kit, and Mitochondrial Isolation kit were obtained from Beyotime. MitoSOX™ Red was from Thermo Fisher. DAPI Fluoromount-G™ was from Yeasen. Mitoquinone (MitoQ) was bought from MCE.

### Cell culture

Bone marrow-derived macrophages (BMDMs) were initially isolated from the bone marrow of 5-week-old C57BL/6J mice. They were subsequently incubated in a RPMI 1640 complete medium supplemented with 30% cultured supernatants of L929 cell lines (ATCC) 29,30 for 5-7 days. To activate NLRP3 inflammasome, BMDMs were induced by 1 μg/mL LPS for 4 h 31 and then stimulated with 10 μM nigericin for 30 min.

Periodontal ligament (PDL) tissues were obtained from extracted teeth following orthodontic treatment to harvest human periodontal ligament stem cells (hPDLSCs), with the informed consent of patients, and then tissues from the middle 1/3 of the root were cut into 1-2mm^2^ pieces. These pieces were covered with coverslips and cultured in a DMEM complete medium. hPDLSCs from passages 3-5 were treated with 10 μg/mL LPS 32 in the ensuing experiments.

### Cell viability assay

Prior to the introduction of Cell counting kit-8 (CCK-8) solution (Dojindo), BMDMs were treated with 1 μg/mL LPS and various doses of Dioscin for 4 h or 24 h, whilst hPDLSCs were stimulated with 10 μg/mL LPS and pre-defined doses of Dioscin for 1 d or 3 d. Lastly, absorbance at 450 nm was recorded.

### RT-qPCR assay

Total RNA from cultured cells or gingival tissues was collected using Trizol. The PrimeScript RT Reagent kit was utilized for reverse transcription. RT-qPCR was carried out using TB Green™ Premix Ex Taq™ II. The above reagents were obtained from Takara and the procedures were followed by the instructions' protocols. The primer sequences used in this study are listed in Table S1.

### ELISA assay

IL-1β (NeoBioscience), tumor necrosis factor (TNF)-α (MultiSciences), and IL-6 (NeoBioscience) Kits were utilized to measure the concentrations of secreted cytokines from BMDMs.

### ROS detection

Intracellular ROS and mtROS levels of BMDMs were measured using ROS Assay Kit and MitoSOX™ Red, respectively. Next, stained cells were evaluated using flow cytometry (Agilent Technologies, USA) or observed under a fluorescence microscope (Leica, Germany) after being stained with Hoechst 33342.

### Immunofluorescence

After rinsing with phosphate buffer saline (PBS), BMDMs were fixed with 4% paraformaldehyde for 10 min. After BMDMs were permeabilized and blocked, they were incubated overnight with anti-apoptosis-associated speck-like protein containing a caspase recruitment domain (ASC) (67824, CST, 1:200) and anti-8-hydroxy-2'-deoxyguanosine (8-OHdG) (BS-1278R, Bioss, 1:200) antibodies, respectively. Thereafter, BMDMs were treated with secondary antibodies in the dark for 1 h and followed by DAPI Fluoromount-G™ staining. Images were visualized under a confocal microscope (LSM800, USA).

### Western blotting

BMDMs were lysed and the collected protein from different groups was separated by sodium dodecyl sulfate-polyacrylamide gel electrophoresis (SDS-PAGE) and then transferred onto polyvinylidene fluoride (PVDF) membranes. After being blocked, the membranes were treated with antibodies overnight, namely anti-NF-κB (8242, CST,1:1000), anti-phospho-NF-κB (3033, CST,1:1000), anti-NLRP3 (15101, CST,1:1000), anti-IL-1β (31202, CST,1:1000), anti-cleaved Caspase-1 (89332, CST, 1:1000), and anti-β-Actin (AF0003, Beyotime,1:1000). After treatment with secondary antibodies for 1 h, blots were captured by a QuantityOne software (Bio-Rad, USA).

### Detection of mtDNA and cytosolic 8-OHdG

Allprep DNA/RNA mini kit (QIAGEN) was utilized to isolate total DNA. mtDNA was analyzed by RT-qPCR. The primer sequences for *non-NUMT*, which is specific for mtDNA, are summarized in Table S1. A mitochondrial Isolation kit was employed to obtain cytosolic fraction. An 8-OHdG quantification kit (EpiQuick) was employed to quantitatively detect 8-OHdG in the DNA of cytosolic fraction.

### K^+^ detection

The levels of supernatant and intracellular K^+^ of BMDMs were quantitatively determined using a K^+^ Assay Kit (Nanjing Jiancheng Bioengineering Institute), and the concentrations of intracellular K^+^ were normalized based on the amount of protein.

### Osteogenic induction and staining

Osteogenesis of hPDLSCs was induced by an osteogenic induction medium. After incubation for 7 d, the expression of alkaline phosphatase (ALP) of hPDLSCs was determined using the ALP assay kit (Beyotime). After 14 and 21 d of culture, hPDLSCs were treated with a BCIP/NBT ALP Kit (Beyotime) for 15 min or alizarin red S (ARS) staining Kit (Beyotime) for 30 min. Finally, images were visualized under a microscope (Leica, Germany).

### Experimental animals

C57BL/6J mice (male, 5-week-old) were provided by Jihui Laboratory (China). Dioscin was dissolved in 0.5% CMNa. After adapting for 1 w, the cervical of the second molar in the left maxillae was tied with 5-0 silk threads. All surgical interventions were performed under anesthesia. After ligation for 1 w, 0.5% CMNa or Dioscin (10 mg/kg) was injected around the second molar once every other day for 2 w. Mice were randomized into the following groups: a) control group, mice received 0.5% CMNa gingival injections without ligation; b) ligature group, consisting of mice with ligature-induced periodontitis receiving gingival injections of 0.5% CMNa; c) ligature + Dioscin group, wherein mice with ligature-induced periodontitis received gingival injections of Dioscin (10 mg/kg). Animal experiments were approved by the Animal Care and Use Committee of the Ninth People's Hospital Affiliated to Shanghai Jiao Tong University School of Medicine.

### Micro-CT analysis

Left maxillae samples were scanned using a micro-CT. The height from the cementoenamel junction (CEJ) to the alveolar bone crest (ABC) of the second molar in each sample was detected to evaluate alveolar bone loss. Moreover, bone volume fraction (BV/TV), bone mineral density (BMD), trabecular thickness (Tb.Th), and trabecular number (Tb.N) were also analyzed.

### Histological evaluation

The left maxillae samples were fixed for 24 h and then decalcified for 4 w. After embedding and sectioning procedures, hematoxylin-eosin (H&E) and IHC were conducted. IHC was performed using anti-IL-1β (ab283818, Abcam, 1:100), anti-8-OHdG (BS-1278R, Bioss, 1:200), and anti-NLRP3 (DF7438, Affinity Biosciences, 1:100) antibodies. Images were visualized under a microscope (Leica, Germany).

### Statistical analysis

Results were shown as mean ± standard deviation (SD). Statistics were conducted on the Prism GraphPad 6.0 and analyzed by the method of one-way ANOVA with Tukey's multiple comparisons test. In this study, *P* < 0.05 was thought statistically significant, labeling not significant as ns, *P* < 0.05 as *, and *P* < 0.01 as **.

## Results

### Dioscin exerts anti-inflammatory activity

**Figure 1:**
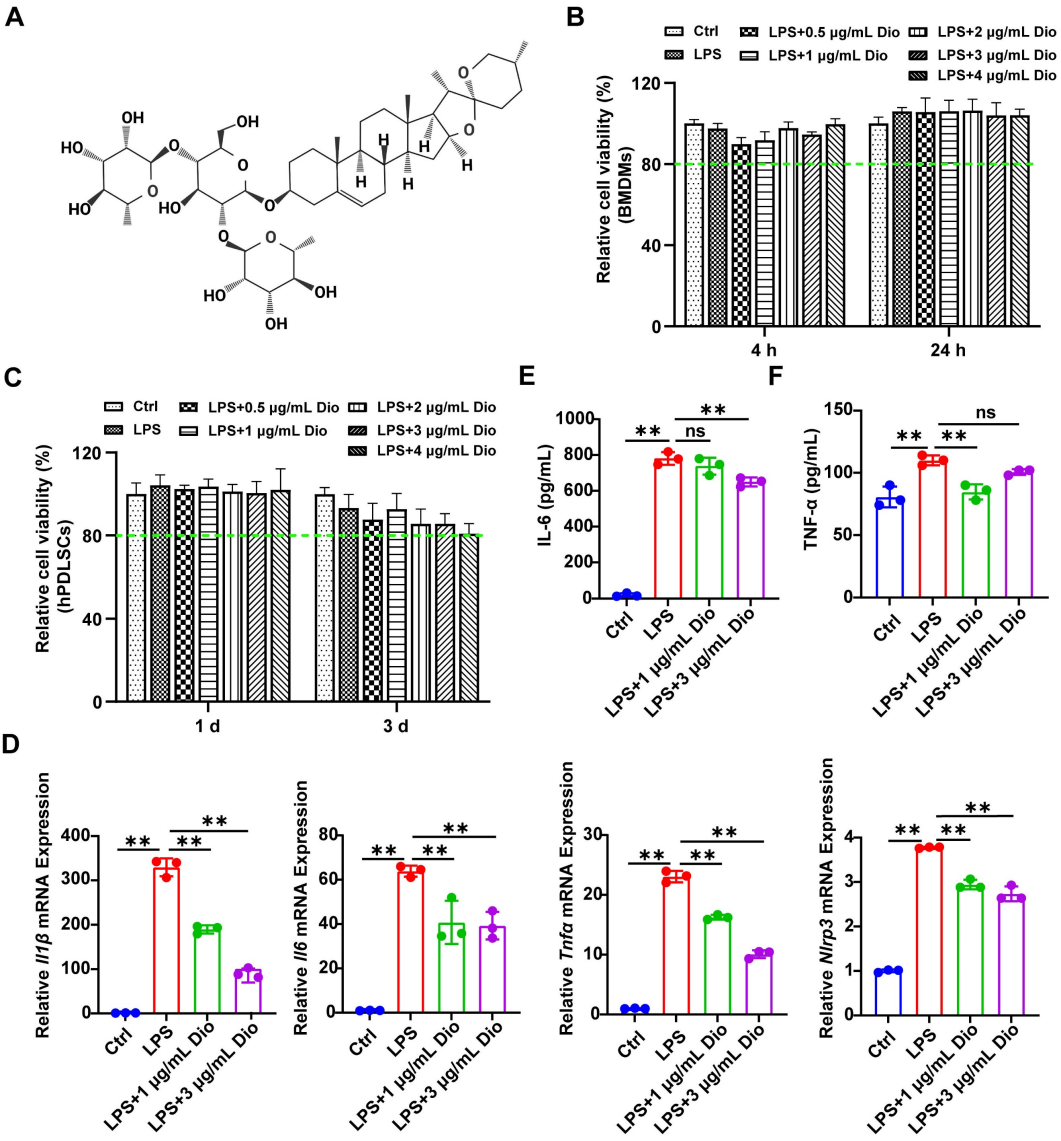
** Dioscin exerts anti-inflammatory activity.** (A) Dioscin structure. (B) Relative cell viability of BMDMs treated with 1 μg/mL LPS and various concentrations of Dioscin (n = 6). (C) Relative cell viability of hPDLSCs treated with 10 μg/mL LPS and various concentrations of Dioscin (n = 6). (D) *Il1β, Il6, Tnfα*, and *Nlrp3* relative mRNA expressions of BMDMs after LPS stimulation for 24 h with or without Dioscin. The concentrations of secreted IL-6 (E) and TNF-α (F) from BMDMs after LPS stimulation for 24 h with or without Dioscin.

Firstly, the cytotoxicity of Dioscin was assessed. The results demonstrated that the cell viability exceeded 80% of the control, indicating that the concentrations of Dioscin lower than 4 μg/mL had very little toxic effect on BMDMs and hPDLSCs (Figure 1B and C). To verify the impact of concentration on the pharmacological activity of Dioscin in vitro, the concentrations of Dioscin utilized in the following experiments were 1 μg/mL and 3 μg/mL. Dioscin inhibited the LPS-induced elevation of *Il1β*,* Il6*,* Tnfα*, and *Nlrp3* mRNA expression (Figure 1D). Consistently, Dioscin curtailed LPS-stimulated secretion of IL-6 (Figure 1E) and TNF-α (Figure 1F) from BMDMs. Taken together, these findings collectively imply that Dioscin could inhibit LPS-induced inflammation without compromising cell viability.

### Dioscin inhibits intracellular ROS production and NF-κB activation

**Figure 2:**
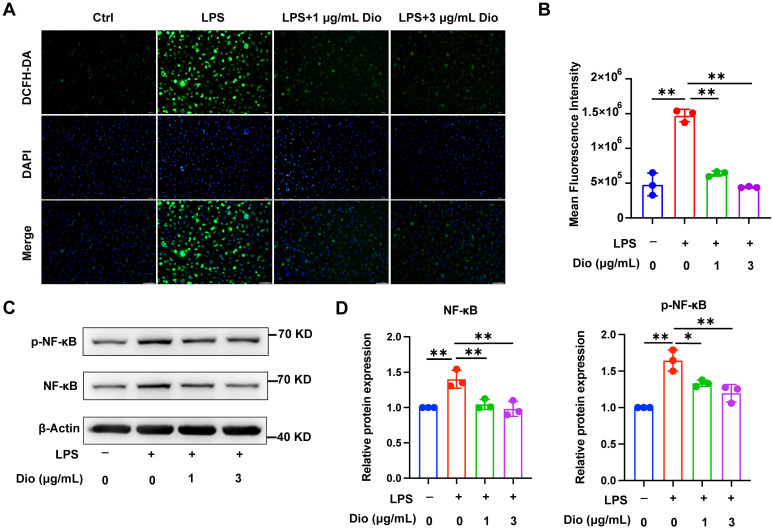
** Dioscin inhibits intracellular ROS production and NF-κB activation.** BMDMs were treated with LPS with or without Dioscin for 4 h. (A) Intracellular ROS was imaged by fluorescence microscope. Scale bars, 50 μm. (B) Intracellular ROS was measured quantitatively using flow cytometry. (C, D) Relative expression of NF-κB and phosphorylation of NF-κB (p-NF-κB).

Ascribed to the persistent inflammatory response to bacterial pathogens, excessive ROS is produced by periodontal immune cells beyond intracellular antioxidant defenses, cumulating in oxidative damage, including protein denaturation and DNA damage, which in turn exacerbates ROS production 33. Intracellular ROS levels in LPS-induced BMDMs were detected using a DCFH-DA probe, and both fluorescence imaging and flow cytometry determined that Dioscin reduced LPS-induced ROS generation (Figure 2A and B). Furthermore, NF-κB is pivotal in regulating the transcription of pro-inflammatory-related genes and *Nlrp3* 34, 35. Meanwhile, Dioscin downregulated LPS-induced phosphorylation of NF-κB (Figure 2C and D). The above data collectively indicate that Dioscin suppresses ROS production and NF-κB activation.

### Dioscin inhibits NLRP3 inflammasome activation

**Figure 3:**
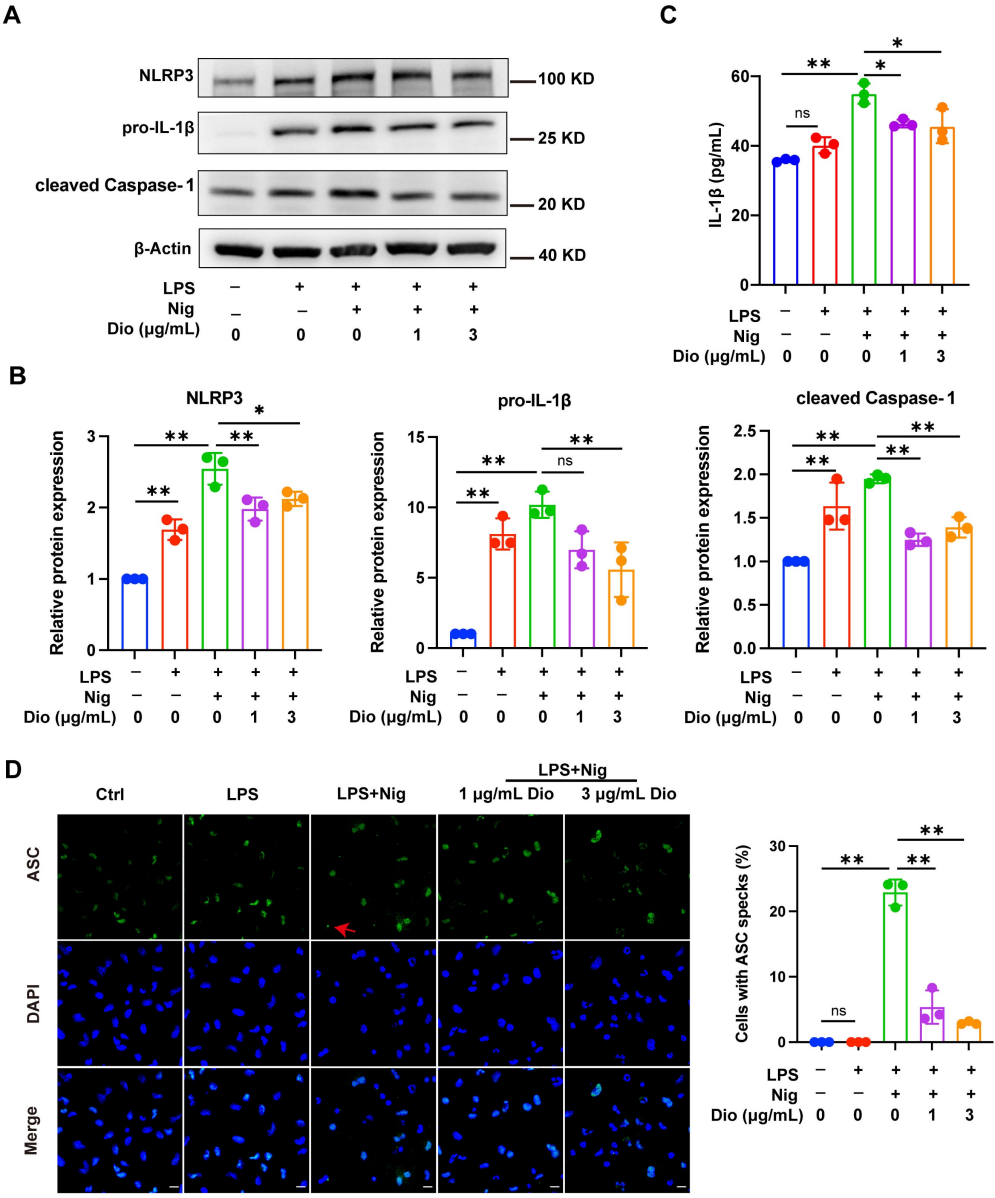
** Dioscin inhibits NLRP3 inflammasome activation.** BMDMs were stimulated by 1 μg/mL LPS for 4 h with or without Dioscin, then incubated with 10 μM nigericin for 30 min. (A, B) Relative protein levels of NLRP3 components. (C) The concentrations of secreted IL-1β. (D) Immunofluorescent staining and analysis of ASC. Scale bars, 10 μm. Red arrows denote ASC specks.

NLRP3 inflammasome, a key factor that governs inflammation, comprises NLRP3, Caspase-1, and ASC. Among them, ASC contributes to the functionalization of Caspase-1. The cleaved Caspase-1 could cleave pro-IL-1β into functional IL-1β 36-38. Nigericin, an NLRP3 agonist, is commonly administered to activate the NLRP3 inflammasome 39. In LPS-primed and nigericin-stimulated macrophages, Dioscin downregulated the protein expression of NLRP3, pro-IL-1β, and cleaved Caspase-1 (Figure 3A and B). Besides, the secretion of IL-1β was also restrained following Dioscin treatment (Figure 3C). Then, it was further investigated whether ASC speck formation, which indicates inflammasome assembly 40, would be affected by Dioscin intervention. As anticipated, Dioscin decreased the proportion of cells with ASC specks (Figure 3D). These findings conjointly indicate that Dioscin could effectively restrain the assembly of NLRP3 inflammasome.

### Dioscin suppresses the production of mtDNA, mtROS, and ox-mtDNA

**Figure 4:**
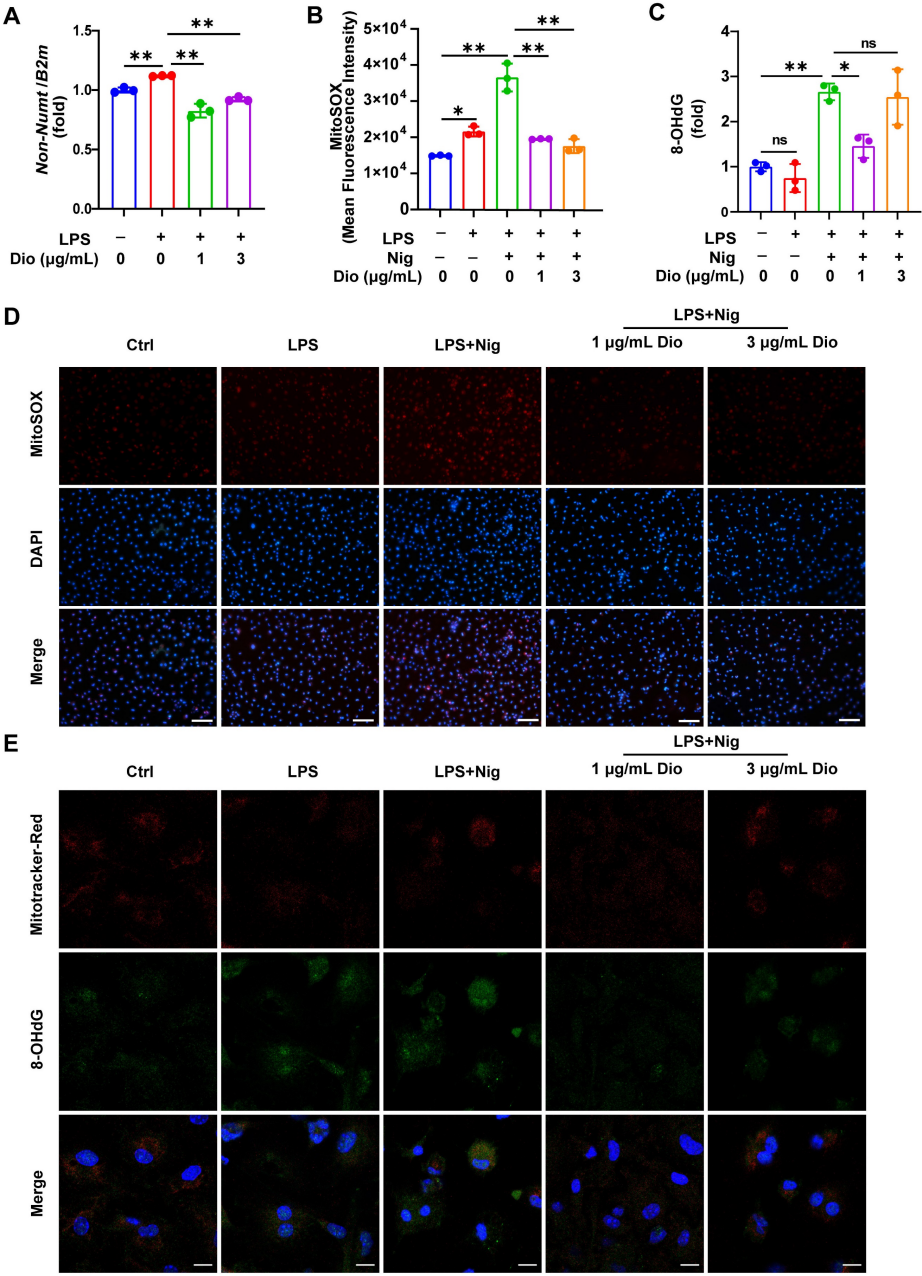
** Dioscin suppresses the production of mtDNA, mtROS, and ox-mtDNA.** BMDMs were stimulated by 1 μg/mL LPS for 4 h with or without Dioscin, then incubated with 10 μM nigericin for 30 min. (A) Relative mtDNA expression. (B, D) mtROS in BMDMs was detected using flow cytometry (B) and fluorescence microscope (D). Scale bars, 50 μm. (C) The amounts of 8-OHdG in cytosolic fraction were assessed using ELISA. (E) Immunofluorescent staining of nuclei (blue), 8-OHdG (green), and mitochondria (red). Scale bars, 10 μm.

Ox-mtDNA has been reported to be a by-product of mtDNA oxidation, and ox-mtDNA contributes to the NLRP3 inflammasome activation 41, 42. Interestingly, the elevation in mtDNA synthesis in LPS-stimulated BMDMs was reversed upon Dioscin intervention (Figure 4A). MitoSOX red probe was used to assess mtROS levels, and both flow cytometry and fluorescence staining results demonstrated that Dioscin inhibited the increased production of mtROS in LPS-primed and nigericin-stimulated macrophages (Figure 4B and D). The ox-mtDNA content was measured using 8-OHdG as an indicator of DNA oxidative damage 41, and the mitochondria were labeled by a Mitotracker-Red probe. The result indicated that Dioscin inhibited the increase in ox-mtDNA content of LPS-primed and nigericin-stimulated macrophages (Figure 4E). Furthermore, Dioscin also alleviated DNA oxidative damage in the cytoplasm (Figure 4C).

These data collectively suggest that Dioscin could inhibit the synthesis of mtDNA and mtROS and decrease the formation of ox-mtDNA.

### Dioscin suppresses K^+^ efflux, which acts upstream of mtROS

K^+^ efflux and excessive generation of ROS could trigger the assembly of NLRP3 inflammasome 43-45. Earlier literature documented that K^+^ efflux facilitated mtROS generation in BMDMs 26, 46. Therefore, the influence of K^+^ efflux on NLRP3 activation and the regulatory role of Dioscin in K^+^ efflux was further investigated.

**Figure 5:**
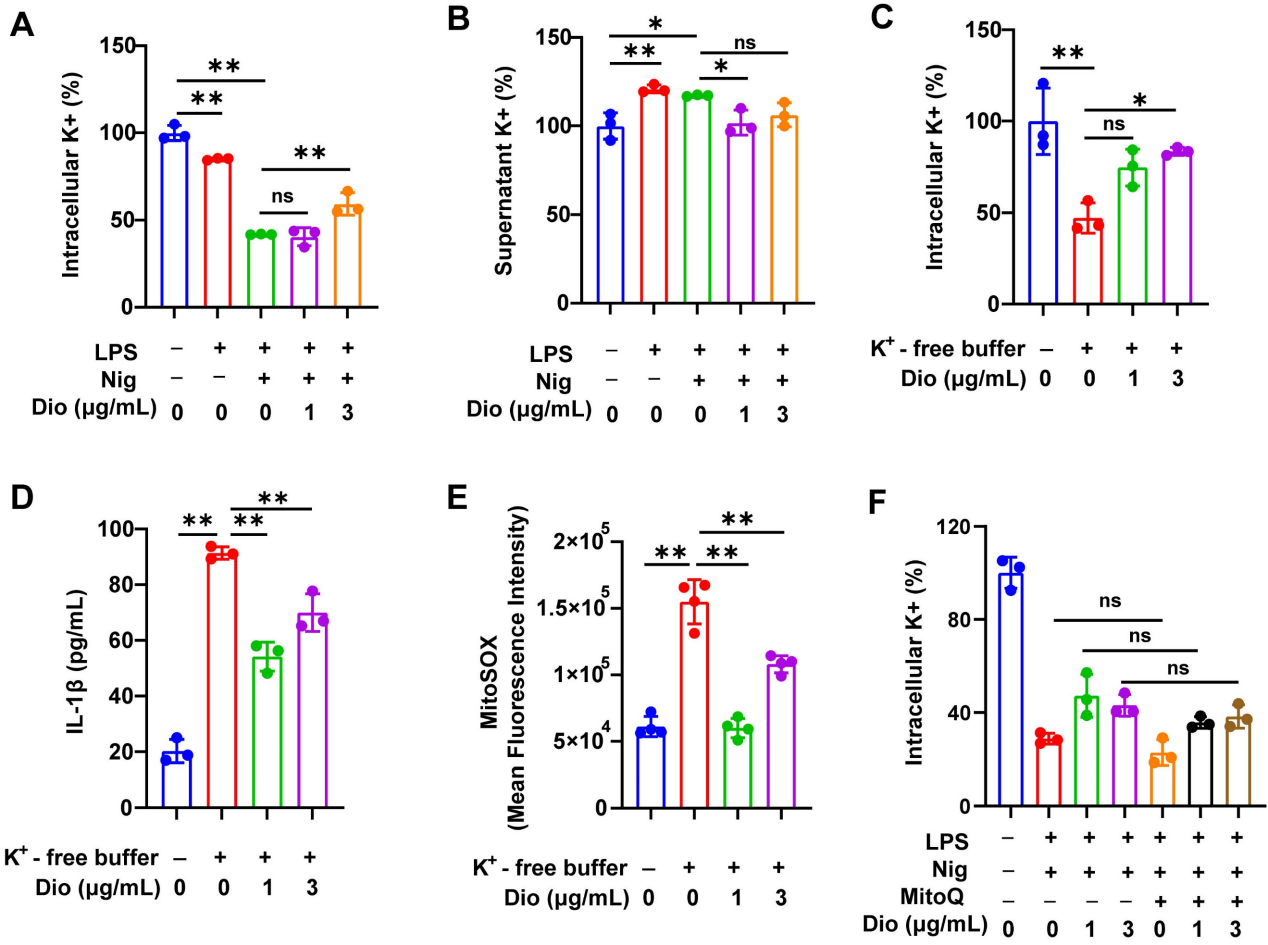
** Dioscin suppresses K^+^ efflux, which acts upstream of mtROS.** (A, B). Intracellular content of K^+^ (A) and supernatant content of K^+^ (B) in BMDMs. (C) Intracellular content of K^+^ in BMDMs induced by K^+^-free buffer (20 mM HEPES (pH 7.4),135 mM NaCl, 1 mM CaCl_2_, 1 mM MgCl_2_, 1 mg/mL BSA, 10 mM glucose) for 4 h with or without Dioscin. (D) The concentrations of secreted IL-1β in K^+^-free buffer-induced BMDMs. (E) mtROS in BMDMs induced by K^+^-free buffer was measured using flow cytometry. (F) Intracellular content of K^+^ in BMDMs. BMDMs were pretreated with Mito Q (500 nM,1 h), stimulated by LPS and nigericin, and treated with Dioscin.

To begin, intracellular K^+^ and supernatant K^+^ levels in LPS- and nigericin-induced BMDMs were examined. The intracellular level of K^+^ was significantly decreased, whereas the concentration of K^+^ in the supernatant was elevated, indicating that the excretion of intracellular K^+^, while Dioscin suppressed this process (Figure 5A and B). Thereafter, BMDMs were transferred into a K^+^-free medium, and the intracellular K^+^ level in BMDMs was significantly reduced (Figure 5C). Notably, K^+^-free buffer dramatically facilitated the secretion of IL-1β, which was reversed by Dioscin (Figure 5D). These data suggest that K^+^ efflux acts upstream in NLRP3 inflammasome activation, and Dioscin may reverse this process by suppressing K^+^ efflux.

The association between K^+^ efflux and mtROS generation was subsequently investigated. The K^+^-free buffer significantly increased the production of mtROS, while Dioscin treatment reversed this effect (Figure 5E). To validate that mtROS acts as an upstream factor in K^+^ efflux, Mito Q, a mitochondria-targeted ROS scavenger, was used to inhibit the elevation in mtROS levels in LPS-primed and nigericin-induced BMDMs, and the results showed that inhibition of mtROS had a minimal impact on K^+^ efflux (Figure 5F), signaling that mtROS may do not function as an upstream regulator of K^+^ efflux.

The above data demonstrate that K^+^ efflux could drive mtROS generation, and activate the NLRP3 inflammasome, whereas Dioscin reversed these effects.

### Dioscin promotes osteogenesis of hPDLSCs under inflammatory conditions

**Figure 6:**
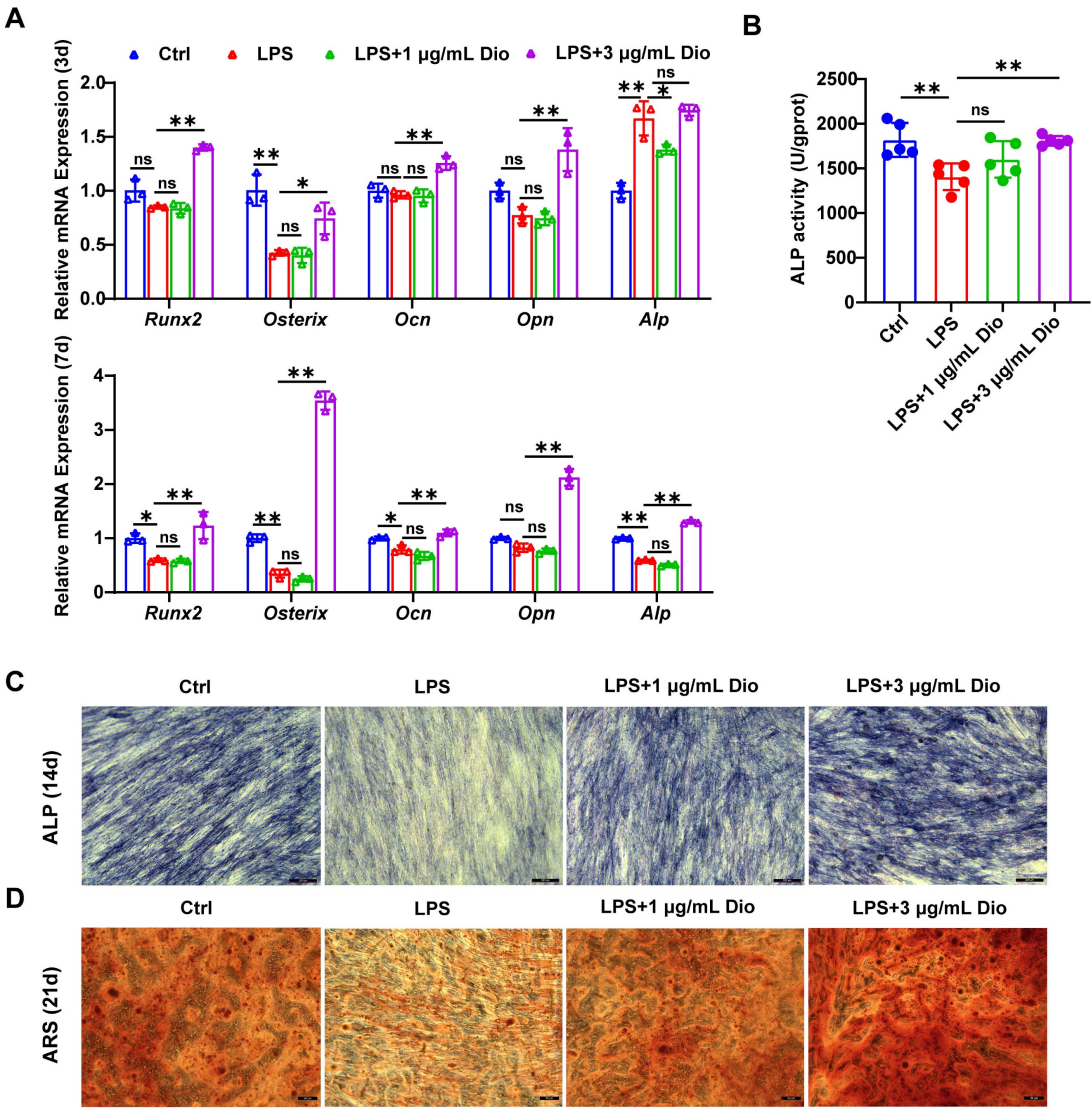
** Dioscin promotes osteogenesis of hPDLSCs under inflammatory conditions.** hPDLSCs were cultured in an osteogenic induction medium and stimulated by 10 μg/mL LPS with or without Dioscin. (A) Relative mRNA levels of osteogenesis genes on day 3 and day 7. (B) ALP activity on day 7. (C) ALP staining of hPDLSCs on day 14. Scale bars, 200 μm. (D) ARS staining of hPDLSCs on day 21. Scale bars, 50 μm.

Under the in vitro LPS-induced inflammatory microenvironment, whether Dioscin affects the osteogenic differentiation of hPDLSCs was explored. The transcription of osteogenic markers, comprising* Runx2*,* Osterix*,* Ocn*, *Opn*, and *Alp*, were measured. After incubation for 3 d, the mRNA levels of *Ocn*, *Opn*,* Osterix*, and *Runx2* in hPDLSCs were upregulated by treatment with 3 μg/mL Dioscin, while 1 μg/mL Dioscin did not promote osteogenic differentiation (Figure 6A). Nevertheless, the mRNA level of *Alp* in LPS-induced hPDLSCs was numerically increased (Figure 6A).

After incubation for 7 d, the transcription of *Ocn*, *Runx2*,* Osterix*, and *Alp* in LPS-induced hPDLSCs was significantly downregulated, whilst the transcription level of these markers was enhanced after treatment with 3 μg/mL Dioscin and almost remained unaltered after the introduction of 1 μg/mL Dioscin (Figure 6A). Consistent with trends in mRNA levels, ALP activity was increased following treatment with 3 μg/mL Dioscin treatment (Figure 6B).

After incubation for 14 and 21 d, ALP staining and ARS staining were carried out, respectively. Notably, the LPS treatment group exhibited shallower ALP staining than the control group and became deeper following Dioscin intervention (Figure 6C). Furthermore, ARS staining displayed fewer mineralized nodules in the LPS treatment group, whereas Dioscin substantially enhanced the formation of mineralized nodules (Figure 6D).

Overall, these data consistently indicate that Dioscin could promote osteogenesis of hPDLSCs under inflammatory conditions.

### Dioscin alleviates alveolar bone loss in periodontitis mice

**Figure 7:**
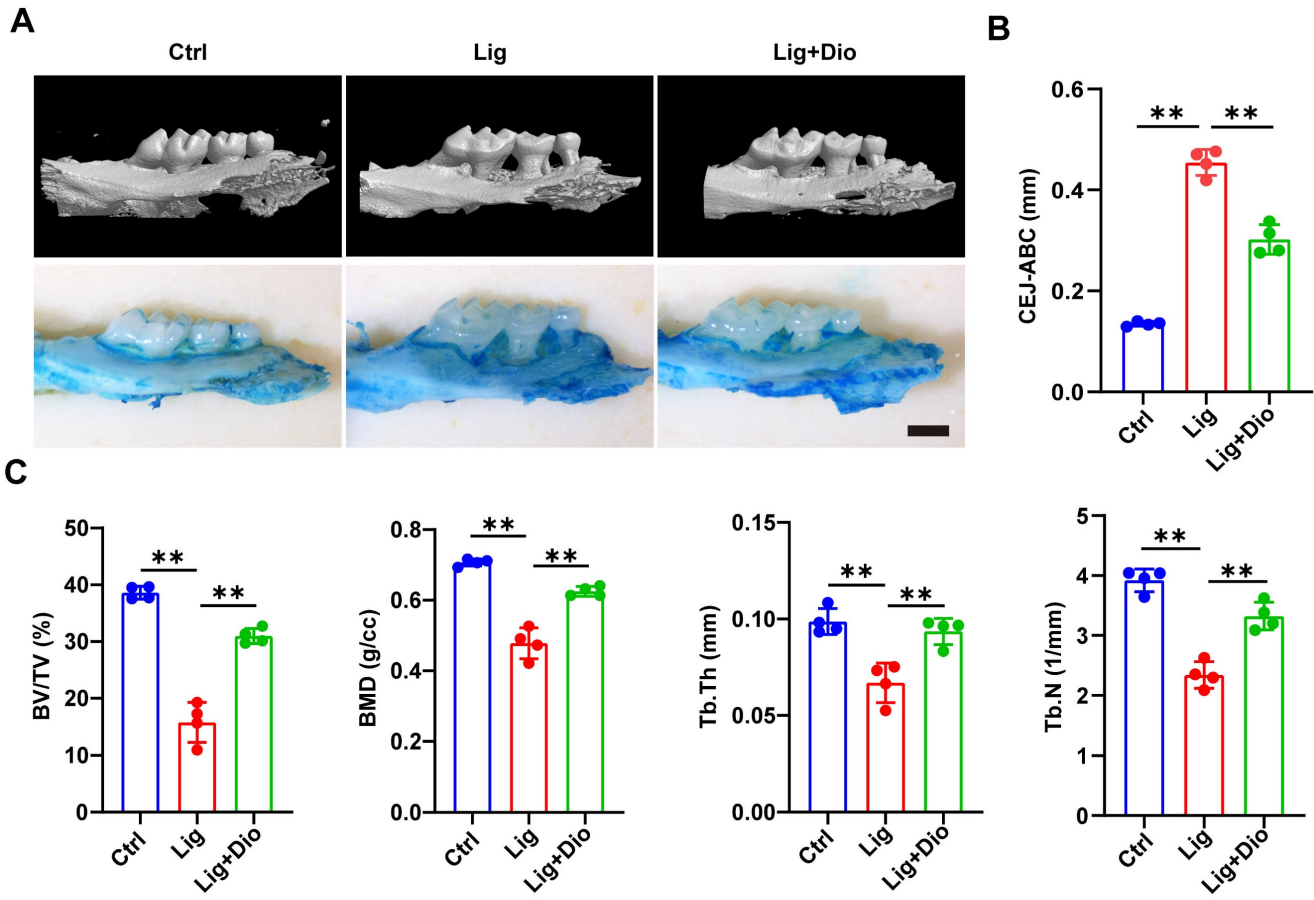
**Dioscin alleviates alveolar bone loss in periodontitis mice.** (A) 3D micro-CT and methylene blue staining images of left maxillae. Scale bars, 2 mm. (B) Assessment of bone loss around the left maxillary second molars. (C) Analysis of bone quality and quantity parameters.

Based on the evidence that Dioscin could inhibit inflammation and promote osteogenesis, mice models of ligation-induced periodontitis were further established. Micro-CT scan and methylene blue staining were employed to analyze the left maxillae of mice in each group (Figure 7A). The height from CEJ to ABC was measured to assess the alveolar bone loss, which was remarkably lower in the Dioscin-intervention group than in the periodontitis group (Figure 7B). This observation was in agreement with the results of methylene blue staining (Figure 7A). To further illustrate the protective role of Dioscin on bone quality and mass, BV/TV, BMD, Tb.Th and Tb.N were analyzed. These parameters were all downregulated in the periodontitis group, reflecting a decrease in bone mass and mineralization. Meanwhile, local injections of Dioscin significantly inhibited bone damage (Figure 7C). These data established that Dioscin is effective in alleviating periodontitis in mice.

### Dioscin suppresses ligation-induced periodontitis

**Figure 8:**
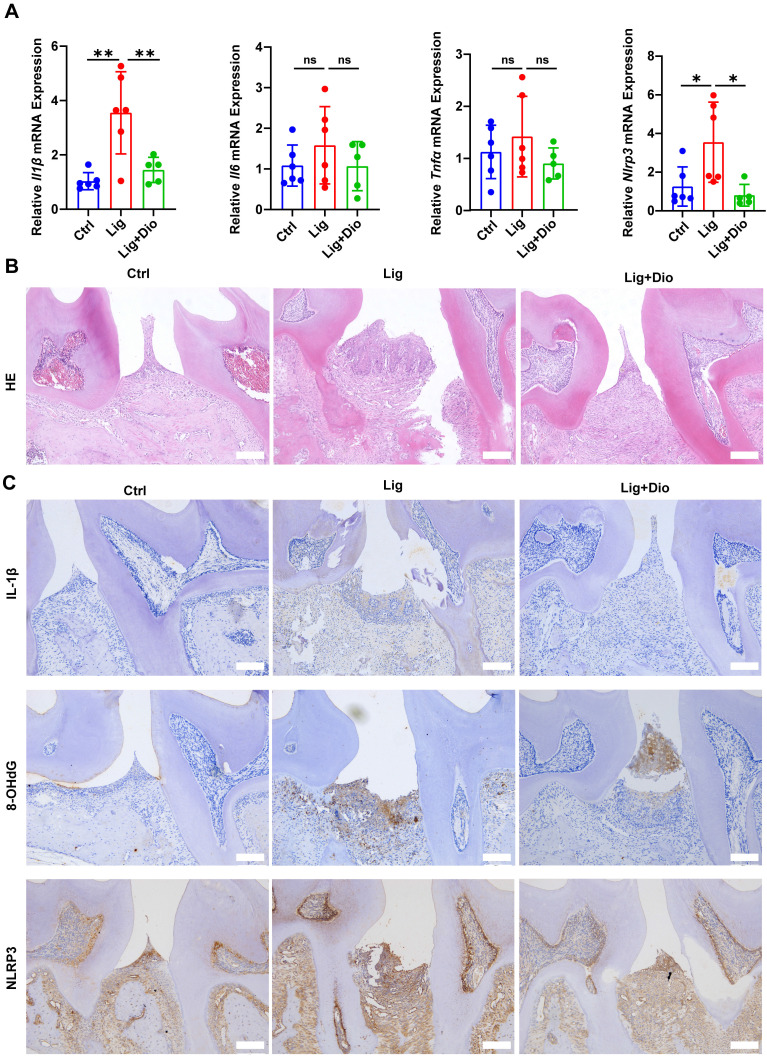
** Dioscin suppresses ligation-induced periodontitis.** (A) Relative mRNA levels of *Il1β*, *Il6*, *Tnfα*, and *Nlrp3* in gingival tissues. (B) HE staining images (n = 3). Scale bars,100 μm. (C) IL-1β, 8-OHdG, and NLRP3 expression in periodontal tissues were determined by IHC staining (n = 3). Scale bars, 100 μm.

The impact of Dioscin on periodontal inflammation was further determined. Of note, ligation-induced increased mRNA expression levels of *Il1β*, *Il6*, *Tnfα*, and *Nlrp3* in gingival tissues were suppressed by treatment with Dioscin (Figure 8A). According to the results of HE staining, inflammatory cell infiltration in the supporting tissue around the second molar in the ligation group was dramatically higher than in the control group, while the inflammatory response was considerably improved after local injections of Dioscin (Figure 8B). Correspondingly, the levels of IL-1β, 8-OHdG, and NLRP3 were substantially upregulated by ligation, which was rescued by treatment with Dioscin (Figure 8C). Thus, Dioscin could relieve periodontal inflammation partly by inhibiting NLRP3 inflammasome.

## Discussion

IL-1β, the maturation and secretion of which is mainly governed by NLRP3 inflammasome, is crucial in bone loss due to inflammation-mediated osteoclastogenesis. Inhibition of NLRP3 inflammasome formation holds the potential to limit alveolar bone loss due to periodontitis. Despite Dioscin being confirmed as an inhibitor of the NLRP3 inflammasome 17, 20, literature on the role of Dioscin in managing periodontitis remains scarce. Therefore, the effectiveness of Dioscin in alleviating periodontitis and the underlying mechanisms were investigated in this study.

To begin, Dioscin significantly curtailed the levels of TNF-α, IL-6, and IL-1β (Figures 1D-F and 3C), which was in line with the results of a prior study that reported that Dioscin downregulated the expression of pro-inflammatory cytokines 47. Apart from IL-1β, IL-6 and TNF-α also contribute to the pathology of periodontitis 48. Indeed, they not only participate in the immune response but also promote RANKL-induced osteoclastogenesis 49-51. Hence, Dioscin was speculated to inhibit periodontitis progression.

Then, the impact of Dioscin on the first step in the activation of NLRP3 inflammasome, namely the priming process, was explored. The results unveiled that the mRNA levels of *Il1β* and *Nlrp3* in LPS-stimulated BMDMs were downregulated by Dioscin, which may be related to the containment of NF-κB phosphorylation (Figure 2C and D). Functionalization of NLRP3 inflammasome requires NLRP3 agonists to activate the assembly of inflammasome components. Exposure to nigericin, an NLRP3 agonist, leads to NLRP3 interacting with ASC and promotes ASC aggregation into a large protein speck, which provides a platform for the activation of Caspase-1. Cleaved Caspase-1 could convert pro-IL-1β to the functional form 36-38. The findings indicated that Dioscin downregulated the expression of cleaved Caspase-1 (Figure 3A and B) and the proportion of cells containing ASC specks (Figure 3D). Likewise, the concentration of secreted IL-1β was decreased following Dioscin treatment (Figure 3C). Therefore, these results insinuate that Dioscin may act as an inhibitor of the NLRP3 inflammasome.

However, the potential pathways by which Dioscin restricts the assembly of NLRP3 inflammasome remain to be elucidated. According to previous studies, mitochondrial damage and mtROS generation are fundamental for NLRP3 inflammasome activation 52-54. In line with an earlier report 28, Dioscin decreased the concentrations of mtROS in LPS-primed and nigericin-stimulated macrophages (Figure 4B and D). Besides, the newly synthesized LPS-induced mtDNA is involved in the activation of NLRP3 41. Meanwhile, mtROS could oxidize mtDNA into ox-mtDNA, which binds NLRP3 to trigger inflammasome activation 52. Herein, Dioscin inhibited the increase in mtDNA synthesis (Figure 4A) and decreased the amounts of ox-mtDNA (Figure 4E), consistent with the containment of NLRP3 inflammasome activation. These discoveries inferred that Dioscin might assist in inhibiting NLRP3 inflammasome assembly by downregulating mtROS and ox-mtDNA generation.

Furthermore, the mechanism by which Dioscin regulates mtROS was investigated. A prior investigation concluded that mtROS generation could be enhanced by impaired ion homeostasis 55. Interestingly, recent studies reported that K^+^ efflux is implicated in mtROS production 26, 46, 56. Specifically, K^+^ efflux could activate NLRP3 inflammasome and augment IL-1β secretion 43, 46, 57. The intracellular concentration of K^+^ was decreased in LPS-primed and nigericin-stimulated BMDMs (Figure 5A), which, as expected, was reversed by Dioscin. To further establish the role of K^+^ efflux on the functionalization of NLRP3 inflammasome, BMDMs were transferred into a K^+^-free buffer. The results indicated that K^+^-free buffer facilitated IL-1β secretion, whereas Dioscin reversed this effect by inhibiting K^+^ efflux (Figure 5C and D). Next, the causal relationship between mtROS and K^+^ efflux was explored. The observations showed that mtROS generation was enhanced in the K^+^-free buffer, which was suppressed by Dioscin intervention (Figure 5E). Then, the influence of mtROS on K^+^ efflux was analyzed, and Mito Q was administered to inhibit mtROS production. However, the results indicated that mtROS did not play a role in regulating K^+^ efflux (Figure 5F), which is aligned with the result of a previous study 44. Therefore, it is postulated that K^+^ efflux acts upstream of mtROS production and further suggests an inhibitory effect of Dioscin on K^+^ efflux.

Topical injection with Dioscin dramatically reduced the mRNA levels of *Nlrp3* and pro-inflammatory genes in gingival tissues (Figure 8A), mitigated the excessive inflammatory response (Figure 8B), and reduced IL-1β, 8-OHdG, and NLRP3 levels in periodontal tissues (Figure 8C). Importantly, micro-CT analysis illustrated that Dioscin intervention rescued ligation-induced alveolar bone resorption both qualitatively and quantitatively (Figure 7A-C). In addition, Dioscin augmented the expression of osteogenic markers and the mineralization in LPS-stimulated hPDLSCs (Figure 6A-D), which partly accounted for the reduced alveolar bone loss following Dioscin treatment in vivo. Overall, our results demonstrated the efficacy of Dioscin for the treatment of periodontitis.

In conclusion, Dioscin effectively relieved periodontal inflammation and inhibited alveolar bone loss. Furthermore, the results inferred that Dioscin could reduce the expression level of pro-inflammatory-related genes and *Nlrp3* via modulating NF-κB in the priming process and inhibit the assembly of NLRP3 inflammasome by regulating K^+^ efflux-mtROS-ox-mtDNA pathway at the activation stage, thereby lowering IL-1β secretion. Taken together, it is postulated that Dioscin might be utilized as an inhibitor of NLRP3 inflammasome for the management of periodontitis and provides a reference for other NLRP3 inflammasome-related diseases.

## Supplementary Material

Supplementary table.
