# Supplementary material for: Dioscin Alleviates Periodontitis by Inhibiting NLRP3 Inflammasome Activation via Regulation of K+ Homeostasis and Mitochondrial Function
Source: Int J Biol Sci. 2024 Jan 27;20(4):1375–88. doi: 10.7150/ijbs.85851 (PMC10878154; doi:10.7150/ijbs.85851)
Supplement: Supplementary file 1 — Supplementary table. [file ijbsv20p1375s1.pdf]

Table S1 The primers used for Real-time quantitative PCR

Genes	Forward Primer (5′ to 3′)	Reverse Primer (5′ to 3′)
m-Il1β	CTTCAGGCAGGCAGTATCACTC	TGCAGTTGTCTAATGGGAACGT
m-Il6	ACAACCACGGCCTTCCCTAC	TCTCATTTCCACGATTTCCCAG
m-Tnfα	CAGGCGGTGCCTATGTCTC	CGATCACCCCGAAGTTCAGTAG
m-Nlrp3	ATTACCCGCCCGAGAAAGG	TCGCAGCAAAGATCCACACAG
m-β-actin	GGTCATCACTATTGGCAACG	ACGGATGTCAACGTCACACT
m-tubulin	GATGCTGCCAATAACTATGCTC	TTGGACTTCTTTCCGTAATCCA
m-non-Numt	CTAGAAACCCCGAAACCAAA	CCAGCTATCACCAAGCTCGT
m-B2m	ATGGGAAGCCGAACATACTG	CAGTCTCAGTGGG GGTGAAT
h-Osterix	CCTGTCCTGTCCTTCTGAGG	ACTCCTGTTCCACTCAGGCT
h-Runx2	TCTTAGAACAAATTCTGCCCTTT	TGCTTTGGTCTTGAAATCACA
h-Ocn	CCCAGGCGCTACCTGTATCAA	GGTCAGCCAACTCGTCACAGTC
h-Opn	CAGTTGTCCCCACAGTAGACAC	GTGATGTCCTCGTCTGTAGCATC
h-Alp	CCACGTCTTCACATTTGGTG	AGACTGCGCCTG GTAGTTGT
h-Gapdh	CACGGCAAATTCCACGGCACAGT	GGGGGCATCAGCAGAAGGAGCAG
